# CD226 Deficiency Alleviates Murine Allergic Rhinitis by Suppressing Group 2 Innate Lymphoid Cell Responses

**DOI:** 10.1155/2022/1756395

**Published:** 2022-07-07

**Authors:** Yang Xie, Yuan Zhang, Tianxiao Zhu, Jingchang Ma, Chujun Duan, Lu Yang, Tingting Wang, Ran Zhuang, Ka Bian, Lianjun Lu

**Affiliations:** ^1^Department of Otorhinolaryngology, Tangdu Hospital, Fourth Military Medical University, #1 Xinsi Road, Xi'an, Shaanxi 710032, China; ^2^Institute of Medical Research, Northwestern Polytechnical University, #127 West Youyi Road, Xi'an, Shaanxi 710032, China; ^3^Department of Periodontology, School of Stomatology, Fourth Military Medical University, #145 West Changle Road, Xi'an, Shaanxi 710032, China; ^4^Department of Immunology, Fourth Military Medical University, #169 West Changle Road, Xi'an, Shaanxi 710032, China

## Abstract

Allergic rhinitis (AR) is an immunoglobulin E-mediated type 2 inflammation of the nasal mucosa that is mainly driven by type 2 helper T cells (Th2) and type 2 innate lymphoid cells (ILC2s). CD226 is a costimulatory molecule associated with inflammatory response and is mainly expressed on T cells, natural killer cells, and monocytes. This study is aimed at elucidating the role of CD226 in allergic inflammatory responses in murine AR using global and CD4^+^ T cell-specific *Cd226* knockout (KO) mice. AR nasal symptoms were assessed based on the frequency of nose rubbing and sneezing. Hematoxylin and eosin and periodic acid–Schiff staining and quantitative real-time PCR methods were used to determine eosinophils, goblet cells, and ILC2-associated mRNA levels in the nasal tissues of mice. CD226 levels on ILC2s were detected using flow cytometry, and an immunofluorescence double staining assay was employed to determine the number of ILC2s in the nasal mucosa. The results showed that global *Cd226* KO mice, but not CD4^+^ T cell-specific *Cd226* KO mice, exhibited attenuated AR nasal symptoms. Eosinophil recruitment, goblet cell proliferation, and Th2-inflammatory cytokines were significantly reduced, which resulted in the alleviation of allergic and inflammatory responses. ILC2s in the murine nasal mucosa expressed higher levels of CD226 after ovalbumin stimulation, and CD226 deficiency led to a reduction in the proportion of nasal ILC2s and ILC2-related inflammatory gene expression. Hence, the effect of CD226 on the AR mouse model may involve the regulation of ILC2 function rather than CD4^+^ T cells.

## 1. Introduction

Allergic rhinitis (AR) is one of the most common chronic diseases, affecting 10%–30% of people globally [1, 2]. The estimated socioeconomic burden of AR management in Asian countries ranges from 30 to 100 billion US dollars [3, 4]. AR is an immunoglobulin E- (IgE-) and type 2 helper T cell- (Th2-) mediated inflammatory response of the nasal mucosa induced by various inhaled allergens. It is characterized by rhinorrhea, nasal condensation, pruritus, and sneezing [5, 6]. In addition to its direct effects, including fatigue, low work efficiency, cognition impairment, and even psychological disorder, it often concomitantly occurs with other allergic comorbidities, such as asthma and conjunctivitis [7].

The first-line treatment for AR, which includes glucocorticoids, antihistamines, and leukotriene receptor antagonists, is less effective than expected, probably because of individual differences and poor patient compliance [7, 8]. In recent years, immunotherapy for AR has been a promising approach [9]. Allergen-specific immunotherapy (AIT) has already been approved for patients who suffer from moderate to severe AR symptoms and who do not get relief from appropriate pharmacotherapy. Nevertheless, the efficacy and safety of AIT need to be further improved [10]. A deeper understanding of the underlying mechanisms of AR may provide novel insights to develop promising strategies for AR treatment.

CD226, also known as DNAM-1 (DNAX accessory molecule-1), is a glycoprotein belonging to the immunoglobulin superfamily and functions as a costimulatory molecule. CD226 is mainly expressed on T cells, natural killer (NK) cells, natural killer T (NKT) cells, monocytes, endothelial cells, mast cells, and eosinophils [11–13]. The interaction of CD226 with its ligands CD155 and CD112 and the competition between CD226 and coinhibitory molecule T cell immunoglobulin and ITIM domain (TIGIT) allow CD226 to be involved in several immunological processes [14, 15]. Previous studies have demonstrated the role of CD226 in antitumor responses, autoimmune diseases, and antivirus responses via immune cell regulation [16–20]. In asthma, CD226-mediated NKT cell activation promotes Th2 cell differentiation, which exacerbates the illness [21]. It is unknown if CD226 modulates other dominant cells, including Th2 cells and type 2 innate lymphoid cells, in airway allergic diseases.

Recent studies have implicated type 2 innate lymphoid cells (ILC2s) as a novel lineage-negative lymphocyte population that produces type 2 cytokines in human allergic disease pathogenesis. ILC2s have been detected in lung, bronchoalveolar lavage, nasal mucosa, nasal polyps, gastrointestinal tract, skin, and blood in humans and mice [22]. However, CD226 expression on ILC2s and its role in the pathogenesis of AR remain unknown. In this study, we mainly evaluated the function of CD226 in the pathophysiology of AR and its function in ILC2s using *Cd226* knockout (KO) mice, which may provide new approaches for AR clinical treatment.

## 2. Materials and Methods

### 2.1. Animals

All experimental procedures were approved by the Scientific Research Ethics Committee of the Fourth Military Medical University (No. 20211008). This study used 8–12-week-old female mice, which were housed in individually ventilated cages and fed with standard laboratory chow and water. Wild type C57BL/6 (WT) mice were purchased from the Animal Center of the Fourth Military Medical University. Global *Cd226* KO mice were kindly provided by Professor Marco Colonna (University of Washington) [23]. *Cd226*-floxed mice (*Cd226*^fl/fl^) were created by Cyagen (Suzhou, China), as we previously reported [24]. *Cd4*-Cre^+/−^ mice were kindly provided by Professor Ning Wu (Tongji Medical College of HUST, China). *Cd226*^fl/fl^ mice were crossed with *Cd4*-Cre mice to generate CD4^+^ T cell-specific *Cd226* KO mice (*Cd226*^∆CD4^). All animals were on a C57BL/6J background.

### 2.2. Ovalbumin- (OVA-) Induced AR Mouse Model

The AR murine model was constructed as previously described, with slight modifications [25]. On days 0, 7, and 14, the mice were injected intraperitoneally with 50 *μ*g of ovalbumin (Sigma–Aldrich, USA) emulsified in 200 *μ*L of sterile phosphate buffered saline (PBS) containing 2 mg of aluminum hydroxide. On days 21–25, 50 *μ*g of OVA dissolved in 40 *μ*L of sterile PBS was delivered intranasally (20 *μ*L per nostril). The mice were sacrificed 24 hours after the final challenge (day 26). The control mice were sensitized and challenged with normal saline (NS).

### 2.3. Nasal Symptom Scores

Within 30 minutes after the final intranasal challenge on day 25, a 10 minute video was recorded. The frequency of nasal rubbing and sneezing behaviors during this period was determined by two blinded independent observers. A continuous nose rubbing behavior was recorded as one event [26].

### 2.4. Routine Blood Tests and Enzyme-Linked Immunosorbent Assay (ELISA)

Blood samples (50 *μ*L) were collected in an anticoagulant tube and analyzed using a CLVC DF-3000Vet automated hematology analyzer (Beijing, China). The resting blood was collected in a centrifuge tube to obtain the serum. Total IgE and histamine levels in the serum were measured using a commercialized ELISA kit (Dakewe Biotech, China; Hengyuan Biotech, China). OVA-specific IgE, IgG1, and IgG2a levels in the serum were determined according to the manufacturer's instructions. In brief, the ELISA plates coated with 10 *μ*g/mL of OVA were incubated with diluted serum samples at 37°C for 1.5 h. Then, antimouse IgE-horseradish peroxidase (HRP) (Invitrogen, USA), antimouse IgG1-HRP (Invitrogen, USA), and antimouse IgG2a-biotin (Invitrogen, USA) were added and incubated for 1 h. For IgG2a-biotin, a subsequent incubation step with HRP-streptavidin (Yeasen, China) was performed. The plates were washed three times between each step. The optical density was determined by measuring the absorbance at 450/595 nm.

### 2.5. Histology

After the mice were sacrificed, the entire nose was isolated and fixed in a 4% paraformaldehyde solution. Subsequently, the nose was decalcified in ethylenediaminetetraacetic acid (EDTA) solution and embedded in paraffin. The tissue sections (4 *μ*m thick) were stained with hematoxylin and eosin (HE) and periodic acid-Schiff (PAS). The thicknesses of the columnar respiratory epithelial cells (CRECs) in the nasal septum were measured using the ImageJ software (National Institutes of Health, Bethesda, MD, USA) [27]. The number of goblet cells was counted per 500 *μ*m of the respiratory epithelium.

The lungs were fixed and embedded in paraffin. Each section (4 *μ*m thick) was stained with HE. The inflammation score was determined based on the infiltration of the inflammatory cells around the bronchus: 0, absent inflammation; 1, bronchus was surrounded by few cells; 2, bronchus was surrounded by inflammatory cells of 1-cell-layer depth; 3, bronchus was surrounded by inflammatory cells of 2–4 cell depth; 4, bronchus was surrounded by inflammatory cells of >4 cell depth. Each slide was evaluated by an average of five individuals [28].

### 2.6. Isolation of Nasal Mucosal ILC2s

The entire nose was isolated, skinned, and minced into small pieces with scissors. Then, the pieces were digested with 1 mg/mL of collagenase IV (DIYIBio, China) and 10 U/mL of DNase I (Beyotime, China) with continuous agitation at 37°C for 40 min. To terminate the digestion process, 10% FBS was added. After filtration through a 70 *μ*m cell filter, the cells were resuspended in 40% Percoll medium (Yeasen, China) and gently added on top of 80% Percoll medium. The cells were then centrifuged at 400 × g for 15 min at room temperature with low acceleration and deceleration rates. The mononuclear cells (MNCs) were collected from the interface between 40% and 80% Percoll medium. The MNCs were then washed twice with ice-cold PBS. ILC2s were identified with flow cytometry using a previously established gating strategy [29, 30].

### 2.7. Quantitative Real-Time PCR (qPCR)

Mouse nasal mucosa was separated from the nasal bone, and the total RNA was extracted with TRIzol reagent (Invitrogen, Carlsbad, CA, USA). First-strand cDNA was synthesized using PrimeScript RT reagent (Takara, Japan). Quantitative real-time PCR was performed on an AriaMx real-time PCR system (Agilent Technologies Inc. Palo Alto, USA) using SYBR (GenStar, China) to analyze the expression of *Il4*, *Il5*, *Il13*, *Il33*, *Cd226*, and *Gata3*. The gene expression levels were normalized against *β*-actin.

### 2.8. Flow Cytometric (FCM) Analysis

For the ILC2 analysis, the isolated MNCs were stained with a cocktail of the following antibodies: CD3e (145-2C11, BioLegend), CD11b (M1/70, eBioscience), CD11c (N418, BioLegend), CD45R (RA3-6B2, eBioscience), CD45 (30-F11, BioLegend), ST2 (RMST2-2, eBioscience), KLRG1 (2F1, eBioscience), and CD226 (10E5, BioLegend). ILC2s were characterized as a subpopulation of Lin (CD3e, CD11b, CD11c, and CD45R)^−^ CD45^+^ cells that expressed KLRG1 and ST2. For intracellular cytokine staining, the MNCs were stimulated with PMA/Ionomycin/BFA for 5 h. After extracellular staining, the cells were fixed, permeabilized, and incubated with anti-IL-5 (TRFK5, eBioscience) and anti-IL-13 (eBioBA, eBioscience) antibodies. For Th2 cell analysis, CD4 (RM4-5, eBioscience), CCR6/CD196 (29-2 L17, BioLegend), and CCR4/CD194 (2G12, BioLegend) antibodies were used. The Th2 cells were defined as CD4^+^ CCR4^+^ CCR6^−^ cells. Absolute counting beads (Invitrogen, USA) were employed to calculate the absolute cell number. FMO (Fluorescence Minus One) and matched isotype control were used as controls. The FCM data were obtained using a spectral cell analyzer (SONY SA3800) and analyzed with the Novoexpress software (Agilent Technologies).

### 2.9. Immunofluorescence Staining

For immunofluorescence double staining, the wrapped nasal mucosa was fixed in 4% neutral buffered formalin and decalcified in an EDTA solution. Slices of 4 *μ*m thickness were obtained and dewaxed in xylene, followed by ethanol gradient dehydration and a PBS wash. Thy-1 (CD90) and ST2 antibodies (Abcam, Cambridge, UK) were used to determine the number of ILC2s. Photo capture was performed using an upright fluorescence microscope (Zeiss, Germany).

### 2.10. Statistical Analysis

The data were presented as mean ± standard error of the mean (SEM). Pairwise comparison was performed using Student's *t*-test. One-way analysis of variance with a Tukey post hoc analysis was used for comparison among multiple groups. *P* < 0.05 was considered to be statistically significant. Statistical analyses were performed using the Prism 9.0 software (Graphpad, La Jolla, CA, USA).

## 3. Results

### 3.1. Lack of CD226 in Mice Ameliorates Allergic Symptoms

**Figure 1:**
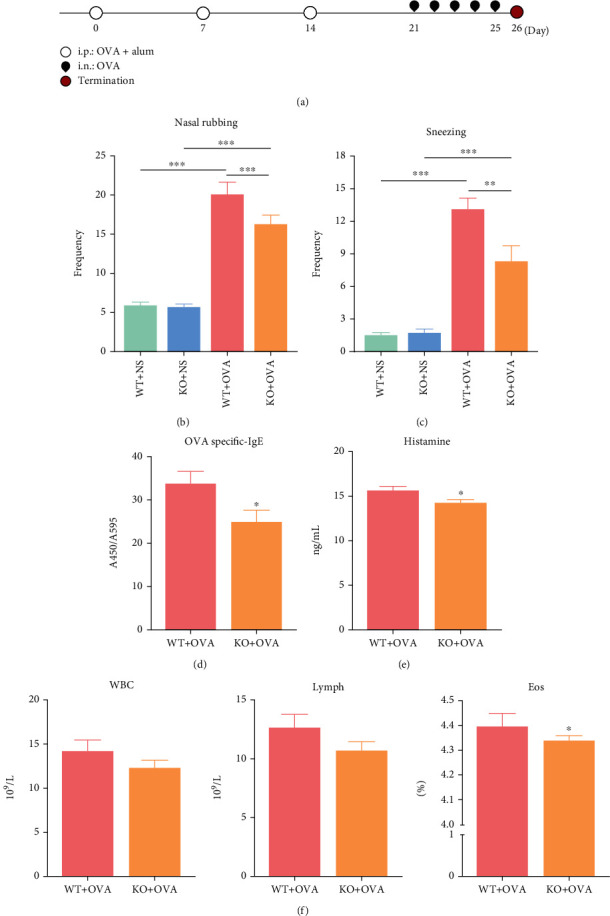
CD226 deficiency significantly attenuates OVA-induced allergic symptoms in AR. (a) Experimental design of the mouse model of AR. (b, c) Frequency of nasal rubbing and sneezing (*n* = 5) within a 10 min period after OVA challenge. (d, e) Serum levels of OVA-specific IgE (*n* = 6) and histamine (*n* = 9) detected using ELISA. (f) Routine blood tests of WBC, Lymph, and Eos (*n* = 7). AR: allergic rhinitis; OVA: ovalbumin; NS: normal saline; IgE: immunoglobulin E; alum: aluminum hydroxide; i.p.: intraperitoneal; i.n.: intranasal; WT: wild type; KO: knockout; WBC: white blood cells; Lymph: lymphocytes; Eos: eosinophils. ^∗^*P* < 0.05, ^∗∗^*P* < 0.01, and ^∗∗∗^*P* < 0.001.

To explore the role of CD226 in AR, we first assessed the OVA-induced AR models in global *Cd226* KO mice and WT mice. The experimental design is presented in Figure 1(a). Nasal allergic symptoms occurred in mice sensitized with OVA and alum (AR group) but not in the NS control group. The effects of CD226 deficiency on the allergic symptoms of mice with OVA-induced AR are presented in Figures 1(b) and 1(c). The frequency of nasal rubbing and sneezing in *Cd226* KO mice was significantly decreased compared with that in WT mice. In addition, the serum levels of OVA-specific IgE and histamine were significantly lower in *Cd226* KO mice compared with those in the WT mice (Figures 1(d) and 1(e)). Furthermore, routine blood tests revealed a reduction in eosinophil proportion in *Cd226* KO mice exposed to OVA, whereas there was no difference in white blood cell and lymphocyte counts between the two groups (Figure 1(f)).

### 3.2. CD226 Deficiency Reduces Upper Respiratory Tract Inflammation in Mice

**Figure 2:**
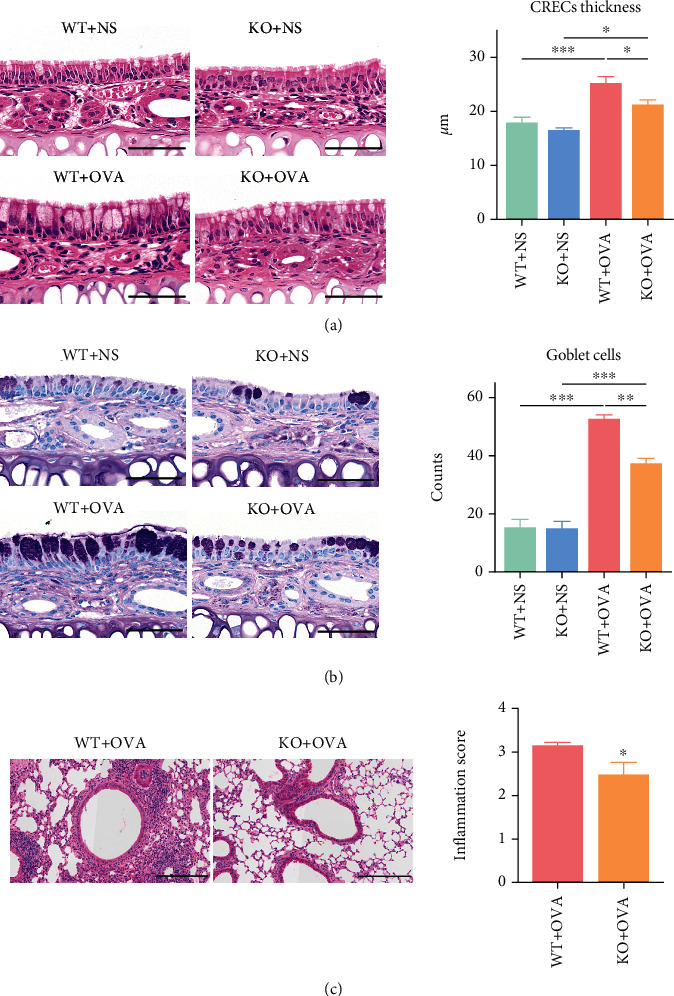
The deficiency of CD226 attenuates OVA-induced inflammation in nasal mucosa and lung in a mouse model of AR. (a) Representative images of HE staining and histopathology. Scale bar = 50 *μ*m. Quantification of thicknesses of CRECs in the nasal septum (*n* = 3). (b) PAS staining and quantification of goblet cells in the nasal mucosa (*n* = 3). Scale bar = 50 *μ*m. (c) Representative images of HE-stained lung sections and evaluation of inflammation scores (*n* = 5). Scale bar = 200 *μ*m. AR: allergic rhinitis; OVA: ovalbumin; NS: normal saline; CRECs: columnar respiratory epithelial cells; HE: hematoxylin and eosin; WT: wild type; KO: knockout. ^∗^*P* < 0.05, ^∗∗^*P* < 0.01, and ^∗∗∗^*P* < 0.001.

The thickness of the nasal mucosa was histologically determined. The thickness of the nasal CRECs was significantly decreased in *Cd226* KO mice compared with WT mice with OVA treatment (Figure 2(a)). Furthermore, PAS staining revealed fewer goblet cells in the nasal mucosa of *Cd226* KO mice with AR (Figure 2(b)). These results indicate that CD226 deficiency attenuates the OVA-induced increase in the mucosal thickness in a mouse model of AR. Expectedly, the lung infiltration and inflammation scores were markedly reduced in *Cd226* KO AR mice compared with WT mice (Figure 2(c)). These results indicate that CD226 might be a positive regulator of AR pathology because CD226 deficiency leads to a remission of AR progress.

### 3.3. CD226 Expression on CD4^+^ T Cells Plays a Limited Role in AR Development

**Figure 3:**
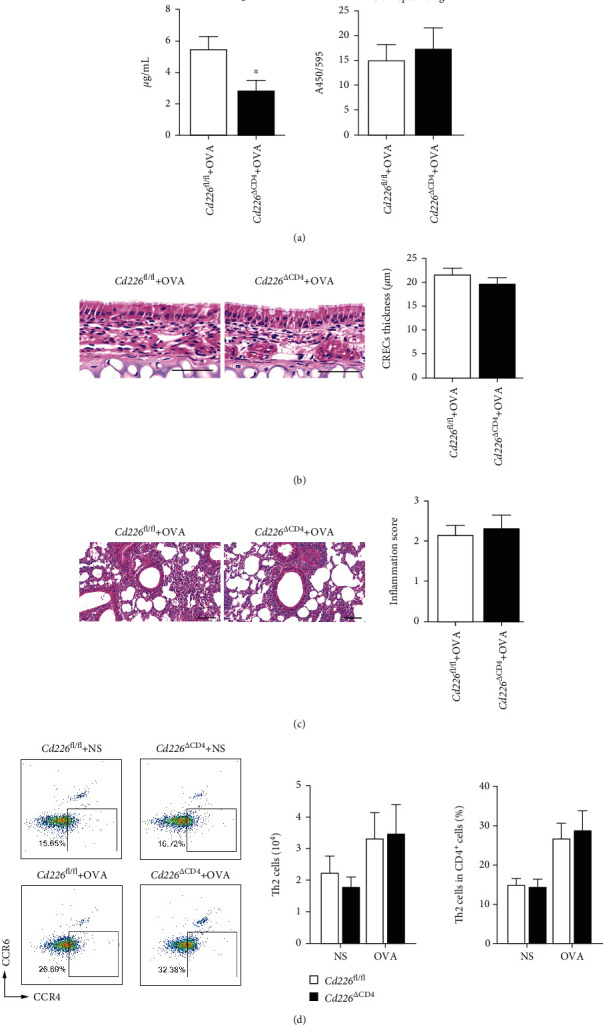
CD226 on CD4^+^ T cells plays a limited role in AR development. (a) Serum levels of IgE and OVA-specific IgE detected using ELISA (*n* = 8). (b, c) Representative images and quantification of HE stained nose and lung sections (*n* = 9). (b) Scale bar = 50 *μ*m and (c) scale bar = 100 *μ*m. (d) Representative images, absolute number, and frequency of nasal Th2 cells (CD4^+^ CCR4^+^ CCR6^−^) were detected using FCM (*n* = 3). AR: allergic rhinitis; OVA: ovalbumin; IgE: immunoglobulin E; ELISA: enzyme-linked immunosorbent assay, ^∗^*P* < 0.05, ^∗∗^*P* < 0.01, and ^∗∗∗^*P* < 0.001.

Given that CD4^+^ T cells are the primary mediators of AR and that CD226 is mainly expressed on T cells, we generated CD4^+^ T cell-specific *Cd226* KO (*Cd226*^∆CD4^) mice to investigate the role of CD226 in CD4^+^ T cells in the pathological process of AR. We found that *Cd226*^∆CD4^ mice exhibited no significant difference in the OVA-specific IgE levels in the serum compared with *Cd226*^fl/fl^ mice. However, the total IgE level was markedly reduced when exposed to OVA challenge (Figure 3(a)). Moreover, mice with a conditional deletion of CD226 in CD4^+^ T cells had a similar phenotype as *Cd226*^fl/fl^ mice on CREC thickness and lung inflammation in the AR process (Figures 3(b) and 3(c)). Subsequently, we evaluated the absolute number and frequency of nasal Th2 cells, which were identified as CD4^+^ CCR4^+^ CCR6^−^ cells [31]. We found that CD4^+^ T cell-specific CD226 deficiency had no effect on the increase in nasal Th2 cells induced by OVA (Figure 3(d)).

### 3.4. CD226 Is Upregulated on the Nasal Mucosal ILC2s during AR

Since AR is characterized by type 2 inflammatory responses, CD226 function on OVA-exposed nasal mucosal ILC2s was investigated. As a lineage-negative population, ILC2s can be detected using flow cytometry via lineage-specific markers to exclude other lymphocytes, including T, B, NK, NKT, and myeloid cells. Furthermore, ILC2s express differential amounts of cell surface molecules, including suppression of tumorigenesis-2 (ST2, also known as IL-33 receptor), IL-2R (CD25), IL-7R (CD127), Thy-1, CD127, inducible T cell costimulatory (ICOS), and killer lectin-like receptor G1 (KLRG1).

**Figure 4:**
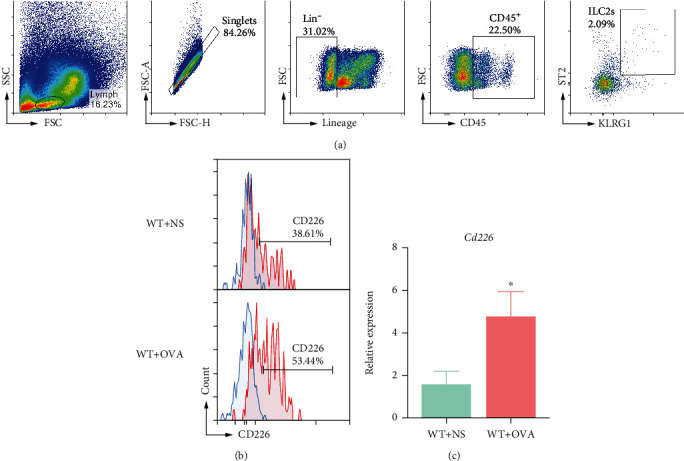
CD226 is inducible on nasal mucosal ILC2s. (a) Gating strategy for the identification of ILC2s (Lin^−^ CD45^+^ ST2^+^ KLRG1^+^). (b) CD226 expression on isolated ILC2s from the nasal mucosa of control and AR mice using FCM assay. (c) CD226 expression in the nasal mucosa of control and AR mice using qPCR assay (*n* = 4). The mice were sensitized against OVA and challenged with OVA at regular intervals; the control mice were sensitized and challenged with only saline. AR: allergic rhinitis; OVA: ovalbumin; NS: normal saline; ILC2s: type 2 innate lymphoid cells; FCM: flow cytometry; qPCR: quantitative real-time PCR; WT: wild type; KO: knockout; ^∗^*P* < 0.05.

As shown in Figure 4(a), ILC2s (Lin^−^ CD45^+^ ST2^+^ KLRG1^+^) from murine nasal mucosa were isolated using a previously reported gating strategy [29]. All gates were set based on FMO or isotype controls. We further measured the CD226 expression levels on the isolated ILC2s from control mice, which were sensitized and challenged with saline and OVA. CD226 was highly expressed on ILC2s in the resting state. OVA challenge further induced CD226 expression, evidenced by a significantly elevated proportion of CD226-expressing ILC2s in the AR group (Figure 4(b)) and higher gene expression of CD226 in the nasal mucosa (Figure 4(c)).

### 3.5. CD226 Deficiency Decreases the ILC2 Proportion in the Nasal Mucosa

**Figure 5:**
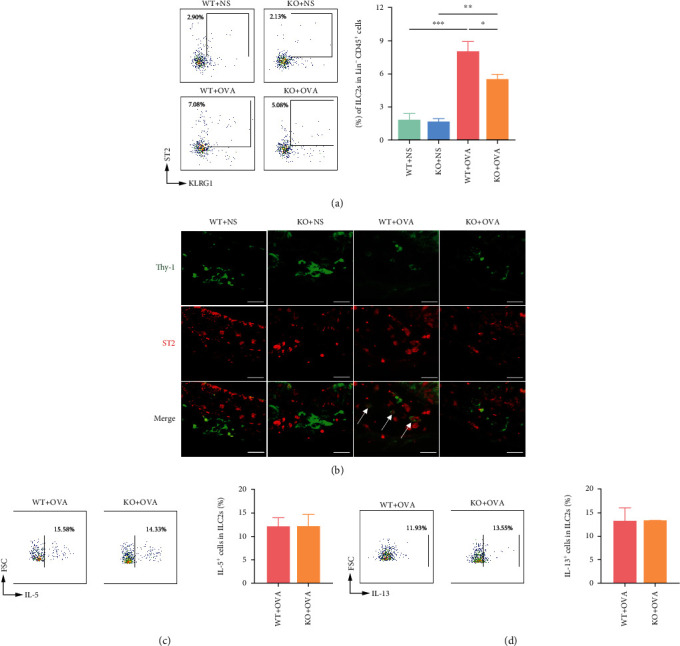
CD226 deficiency reduced the proportion of ILC2 in the nasal mucosa in AR. (a) Mononuclear cells were isolated from the nasal mucosa of WT and *Cd226* KO mice. The proportion of ILC2s (Lin^−^ CD45^+^ ST2^+^ KLRG1^+^) among Lin^−^ CD45^+^ cells (*n* = 3). (b) Representative images of immunofluorescence staining with ST2 and Thy-1 antibodies. ILC2s were positively stained for both ST2 and Thy-1. The white arrows represent ILC2s. Each experiment was repeated three independent times. Scale bar = 25 *μ*m. (c, d) The proportions of (c) IL-5^+^ and (d) IL-13^+^ cells in ILC2s (*n* = 3). AR: allergic rhinitis; OVA: ovalbumin; NS: normal saline; ILC2s: type 2 innate lymphoid cells; WT: wild type; KO: knockout. ^∗^*P* < 0.05, ^∗∗^*P* < 0.01, and ^∗∗∗^*P* < 0.001.

ILC2s were recruited to the nasal mucosa, and the proportion of ILC2s significantly increased with the severity of AR. FCM analysis demonstrated that the proportion of ILC2s in the AR group was significantly higher than that in the NS group. In contrast, CD226 deficiency dramatically decreased the proportion of ILC2s in the nasal mucosa compared with WT mice (Figure 5(a)). Additionally, double immunofluorescence staining for Thy-1 and ST2 was used to detect the number of ILC2s in nasal mucosa tissues. The results similarly showed that the amounts of ILC2s in the OVA-administered groups were significantly higher than those in the NS groups, and that *Cd226* KO significantly attenuated the immunoreactivity of Thy-1 and ST2 in the WT mice with AR (Figure 5(b)). The proportions of IL-5^+^ and IL-13^+^ ILC2s showed no significant difference in these two groups after OVA treatment (Figures 5(c) and 5(d)).

### 3.6. The Expression of Type 2-Related Genes Was Reduced in the Nasal Mucosa of CD226 KO Mice with AR

**Figure 6:**
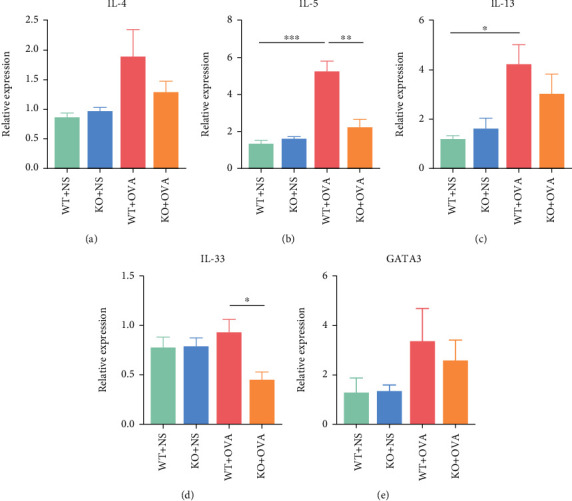
The expression levels of mRNA in mice nasal mucosa using qPCR. The expression levels of (a) *Il4*, (b) *Il5*, (c) *Il13*, (d) *Il33*, and (e) *Gata3* in the nasal mucosa (*n* = 3 in NS groups; *n* = 5 in OVA groups). All data represent mean ± SEM. AR: allergic rhinitis; OVA: ovalbumin; NS: normal saline. ^∗^*P* < 0.05, ^∗∗^*P* < 0.01, and ^∗∗∗^*P* < 0.001.

ILC2 function is mainly associated with the production of type 2 cytokines and related genes, such as *Il4*, *Il5*, *Il13*, *Il33*, and *Gata3*. The mRNA levels of type 2-related genes in the nasal mucosa tissues from WT and *Cd226* KO mice, with or without OVA challenge, were detected using qPCR. As shown in Figure 6, *Il5* and *Il13* levels were significantly increased in mice with OVA-induced AR compared with NS groups; in AR mice, CD226 deficiency significantly decreased the *Il5* and *Il33* levels compared with those of the WT mice (Figure 6). Although no significant alterations were observed in the other genes, they all showed similar tendencies among the groups. These results suggest that knocking out *Cd226* ameliorates the allergic response of AR partially because of decreased type 2-related gene expression.

## 4. Discussion

Allergic airway diseases, such as asthma and AR, are characterized by Th2 inflammation [1, 2, 5]. ILC2s are a newly discovered and significant Th2 cell type of the innate immune system. ILC2s are predominantly distributed in the mucosal tissues, such as lung, intestine, skin, and adipose tissue [32, 33]. ILC2s do not express antigen-specific receptors; however, similar to Th2 cells, they produce type 2 cytokines, including IL-4, IL-5, IL-9, and IL-13, when exposed to epithelium-derived cytokines, such as IL-33, IL-25, and thymic stromal lymphopoietin [33–35]. Type 2 cytokines thus initiate the differentiation of B cells into plasma cells producing antigen-specific IgE, subsequently increasing vascular permeability, degranulation of mast cells, and infiltration of basophils, eosinophils, and neutrophils.

Neither human nor mouse ILC2s express lineage markers; nonetheless, ILC2s in the normal physiological state express surface molecules, such as CD45, Thy-1, c-kit, and MHC-II. Activated ILC2s can express KLRG1 and ST2 at high levels according to their tissue location and activity status [36, 37]. The role of ILC2s in AR remains uncertain, and their role in allergic diseases should be elucidated to understand the relationship between allergic reactions and the immune system [33].

In this study, we showed for the first time that the costimulatory molecule CD226 affected the pathophysiology and progression of AR in a mouse model. The AR nasal symptoms, number of goblet cells, inflammation scores of the nasal mucosa and the lung, and OVA-specific IgE in the serum of the *Cd226* KO group were significantly lower than those of the WT group. Although global *Cd226* deficiency ameliorates allergic symptoms and airway inflammation, the symptoms were not significantly improved in the CD4^+^ T cell-specific *Cd226* KO mice. Therefore, we speculated that CD226 may play a crucial role in modulating local-resident ILC2s (lung and nasal tissue), which drive innate type 2 inflammation, thereby affecting AR progression.

Accumulating evidence indicates that immune checkpoint molecules on ILC2s are potential therapeutic targets for allergic diseases [38]. In this study, we found that the costimulatory molecule CD226 may act as a positive regulator of ILC2s in allergic inflammation. The administration of OVA markedly increased CD226 expression on ILC2s in the nasal mucosal of mice. Loss of CD226 significantly reduced the proportion of ILC2s and alleviated ILC2-driven inflammation in the local tissue. The expression levels of the Th2-type cytokines *Il4*, *Il5*, and *Il13* and ILC2-related genes, *Il33* and *Gata3*, in the nasal mucosa of the AR group were significantly higher than those in the NS group; CD226 loss reversed this tendency. These findings suggest that the loss of CD226 alleviated the Th2 cell-mediated inflammatory response of AR dominated by ILC2s, which may aid in the development of novel therapeutic agents for AR.

However, since *Cd226* whole-body knockout mice and CD4^+^ T cell-specific *Cd226* KO mice were mainly applied in this study, direct evidence indicating the effects of ILC2-derived CD226 on the pathogenesis of AR is still lacking. To further clarify the function of ILC2s, appropriate animal models, such as adoptive ILC2 transfer and ILC2-specific gene knockout mice, are needed. For example, Helou et al. adoptively transferred in vitro cultured human ILC2s into *Rag2*^−/−^*Il2rg*^−/−^ mice, followed by PD-1 agonist administration, to study the role of PD-1 in human ILC2s [39]; Howard *et al.* sorted ILC2s from IL-33-treated PD-1 knockout mice and transferred them into *Rag2*^−/−^*Il2rg*^−/−^ mice to evaluate PD-1 function in ILC2s [40]. Moreover, Nussbaum *et al.* generated reporter mice Red5 (recombinase-expressing detector for IL-5, R5) [41], and the R5/+ mice have been widely used to construct ILC2-specific gene deletion models [42, 43]. Hence, further studies are necessary to ascertain the intrinsic role of CD226 in ILC2s.

Furthermore, CD226 and its ligand nectin-2 (CD112) are expressed on mast cells, eosinophils, and other major effector cells in allergic processes. Studies have shown that CD226 synergizes with Fc*ε*RI on mast cells, and that its engagement augments degranulation via a pathway involving Fyn, LAT, and PLC*γ* [44, 45]. Our findings were observed in global *Cd226* KO mice and CD4^+^ T cell-specific *Cd226* KO mice; however, we did not rule out the possibility that CD226 on other immune cells may play a critical role in the development of allergic reactions.

## 5. Conclusions

In this study, we found that deficiency of CD226 relieved AR symptoms and inhibited immune response of AR mice. ILC2s play an important role in the occurrence and development of AR, and loss of costimulatory molecule CD226 reduced the number of ILC2s and inhibited the ILC2-related cytokine expression. Our data provide new information on the mechanism of action of ILC2s in AR, but the physiological significance of CD226 for ILC2 development and function needs to be further explored.

## Data Availability

The data used to support the findings of this study are available from the corresponding authors upon request.
